# Directed self-assembly of block copolymer films on atomically-thin graphene chemical patterns

**DOI:** 10.1038/srep31407

**Published:** 2016-08-16

**Authors:** Tzu-Hsuan Chang, Shisheng Xiong, Robert M. Jacobberger, Solomon Mikael, Hyo Seon Suh, Chi-Chun Liu, Dalong Geng, Xudong Wang, Michael S. Arnold, Zhenqiang Ma, Paul F. Nealey

**Affiliations:** 1 Department of Electrical and Computer Engineering, University of Wisconsin-Madison, Madison, Wisconsin 53706, United States; 2 Institute for Molecular Engineering, University of Chicago, Illinois 60637, United States; 3 Department of Materials Science and Engineering, University of Wisconsin-Madison, Madison, Wisconsin 53706, United States; 4 IBM Albany NanoTech, Albany, New York 12203, United States

## Abstract

Directed self-assembly of block copolymers is a scalable method to fabricate well-ordered patterns over the wafer scale with feature sizes below the resolution of conventional lithography. Typically, lithographically-defined prepatterns with varying chemical contrast are used to rationally guide the assembly of block copolymers. The directed self-assembly to obtain accurate registration and alignment is largely influenced by the assembly kinetics. Furthermore, a considerably broad processing window is favored for industrial manufacturing. Using an atomically-thin layer of graphene on germanium, after two simple processing steps, we create a novel chemical pattern to direct the assembly of polystyrene-block-poly(methyl methacrylate). Faster assembly kinetics are observed on graphene/germanium chemical patterns than on conventional chemical patterns based on polymer mats and brushes. This new chemical pattern allows for assembly on a wide range of guiding periods and along designed 90° bending structures. We also achieve density multiplication by a factor of 10, greatly enhancing the pattern resolution. The rapid assembly kinetics, minimal topography, and broad processing window demonstrate the advantages of inorganic chemical patterns composed of hard surfaces.

The self-assembly of block copolymers through microphase separation yields patterns with various morphologies and feature sizes ranging from 3 to 100 nm^1^,^2^,^3^,^4^,^5^. Defect-free and well-registered block copolymer domains can be created by directed self-assembly on patterned topographic features, known as graphoepitaxy^6^,^7^,^8^, or on patterns with controlled chemical contrast, known as chemoepitaxy^9^,^10^,^11^,^12^,^13^. Directed self-assembly can improve the line-edge roughness of the lithographically-defined template^14^, correct defects in the underlying template via a self-healing mechanism^15^, and enhance the pattern resolution compared to the periodicity of the template via density multiplication^13^,^16^.

The Liu-Nealey (LiNe) flow is a paradigm of chemoepitaxy, as it seamlessly integrates with state-of-the-art 193 nm immersion lithography^17^,^18^. Chemical patterns based on cross-linkable polystyrene (X-PS) are commonly used in the LiNe flow because X-PS preferentially wets the polystyrene (PS) domains in the widely studied polystyrene-block-poly(methyl methacrylate) (PS-*b*-PMMA) system^19^. After the X-PS mat (6–8 nm in thickness) is patterned to form line-space arrays, the guiding stripes are typically trimmed with a plasma etch. Then a random copolymer brush with hydroxyl groups is grafted into the void spaces between the guiding lines, followed with solvent rinsing to remove excess brush material. This process, known as backfilling, controls the chemistry of the background regions to minimize the interfacial energy between the block copolymer film and the patterned surface, and reduces the step height of the chemical pattern^19^. However, these processing steps can complicate the chemical pattern. For example, plasma trimming oxidizes the sidewalls of the X-PS stripes and forms a trapezoidal shape, consequently changing the guiding scheme of the chemical pattern into one that has three tones^20^,^21^. Furthermore, the grafted brush layer can cause interdigitation of the polymer chains with the random copolymer brush, decreasing their diffusivity and resulting in slowed chain dynamics during assembly^22^.

Alternatively, if a material that wets PS or PMMA is patterned on a neutral surface, only lithography and plasma etching are needed to prepare the chemical contrast pattern. Here, monolayer graphene grown on a germanium wafer is proposed as an appealing template to perform directed self-assembly. Graphene has similar wetting behavior as an X-PS mat^23^,^24^, but is a single atom in thickness, forming nearly no topography and providing a smooth, flat, and rigid surface for the assembly of block copolymers. Graphene also has a well-defined step height and its inert surface interacts relatively weakly with block copolymers, which may enhance the mobility of polymer chains during segregation and result in more rapid assembly kinetics. Due to its sp^2^ bonded lattice with carbon-carbon bond distance of 1.42 Å, graphene also has high mechanical strength^25^, thermal stability^26^, chemical inertness, and impermeability^27^, making it a robust template that is compatible with a broad range of manufacturing conditions. Continuous graphene films can be deposited over the wafer scale via chemical vapor deposition (CVD)^28^,^29^,^30^, which ensures uniform surface chemistry and monolayer thickness over large areas. In previous work, chemically-modified graphene has been used as a suitable surface for the self-assembly of block copolymers into random fingerprint features^31^,^32^. However, the use of a two-dimensional material as a template for directed assembly of block copolymers into ordered patterns would extend the capabilities of block copolymer lithography.

In this work, we demonstrate that atomically-thin graphene stripe arrays can be used as chemical patterns for directed assembly of block copolymers, resulting in well-registered lamellae patterns with complex architectures. The graphene/germanium chemical patterns enable block copolymer assembly on templates with incommensurate periods and in 90° bending structures. Faster assembly dynamics are observed on graphene/germanium templates than on conventional patterns based on polymer mats and brushes. Density multiplication by a factor of 10 is also demonstrated, which means that the period of the assembled block copolymer is one-tenth the template periodicity. These results indicate that graphene/germanium chemical patterns enable robust, reproducible directed self-assembly of block copolymers.

## Results and Discussion

### Directed assembly on graphene chemical patterns

**Figure 1:**
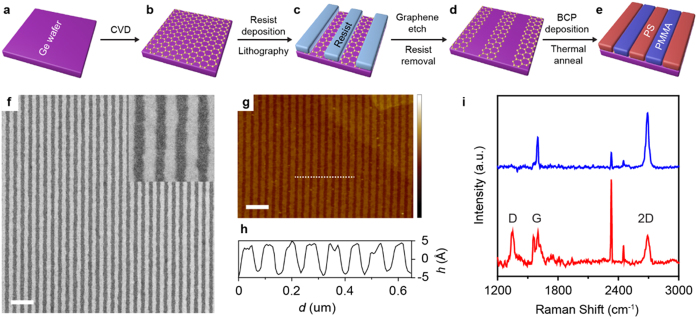
Characterization of the graphene/germanium chemical pattern. (**a–e**) Process flow of the block copolymer directed assembly on graphene/germanium chemical patterns. Graphene is grown directly on germanium via CVD (**a**,**b**). Resist is spin-coated onto the graphene and patterned into stripe arrays (**c**). The exposed graphene is etched using a reactive ion plasma and the resist is removed in solvents and via thermal annealing, resulting in a graphene/germanium stripe array (**d**). The block copolymer is spin-coated on the chemical pattern and thermally annealed to direct assembly (**e**). (**f**) SEM image of a graphene stripe array (bright lines) on germanium (dark lines). Inset shows a magnified image highlighting the edges of the graphene stripes. (**g**,**h**) AFM topographic map (**g**) and height, *h*, profile plotted against surface distance, *d*, (**h**) along the dashed line in (**g)**. Scale bars in (**f**,**g)** are 250 nm. Height scale bar in (**g)** is 8 nm. (**i)**, Raman spectrum of an as-synthesized monolayer graphene film on germanium (top blue spectrum) and a graphene stripe array on germanium (bottom red spectrum). The sharp peaks at ~1555 and ~2330 cm^−1^ correspond to ambient oxygen and nitrogen, respectively.

First, we show that chemical patterns consisting of graphene guiding stripes on germanium can direct the assembly of block copolymers into straight, parallel lamellae structures. In this process, which is shown schematically in Fig. 1a–e, continuous monolayer graphene films are grown directly on germanium wafers via CVD (Fig. 1a,b). Scanning electron microscopy (SEM) and atomic force microscopy (AFM) show that the graphene surface is uniform with low roughness of 1–2 nm over 10 × 10 μm^2^ (Supplementary Fig. S1). The underlying germanium surface consists of terraces and steps, which are predominately one atomic layer in height and are induced during synthesis, and the graphene film contains sparse wrinkles that are formed during post-growth cooling. Raman spectroscopy (Fig. 1i, top blue spectrum) confirms that the graphene is predominately a single layer in thickness, as indicated by the integrated 2D:G ratio of ~4 and the Raman 2D full-width-at-half-maximum of ~26 cm^−1^ 33. The negligible Raman D band intensity indicates that the graphene is of high quality, with a relatively low defect density^34^.

Line arrays are patterned into a poly(methyl methacrylate) (PMMA) mask over the continuous graphene monolayer films using electron-beam lithography (Fig. 1c). The exposed graphene regions are subsequently etched with reactive oxygen ion plasma, resulting in the formation of isolated graphene guiding stripes (Fig. 1d,f–h). Mild etching conditions (10 sccm O_2_, 50 W, 10 mtorr, 1–2 s) are used to ensure that the germanium surface is not etched and that the single-atom step height of the graphene stripes is preserved (Fig. 1g,h). The samples are sonicated and rinsed in chlorobenzene and toluene and then annealed at 350–400 °C at ~10^−5^ torr to remove residue and contamination introduced during patterning. This cleaning ensures a pristine, atomically-thin chemical pattern, which increases the fidelity and reproducibility of the directed block copolymer assembly. The height profile along the graphene stripe array (Fig. 1g,h) indicates that the average step height of the chemical pattern is ~5 Å, consistent with a single layer of graphene. After etching, the Raman D:G ratio increases to ~1 (Fig. 1i, bottom red spectrum), consistent with increased graphene edge density due to ribbon formation.

**Figure 2:**
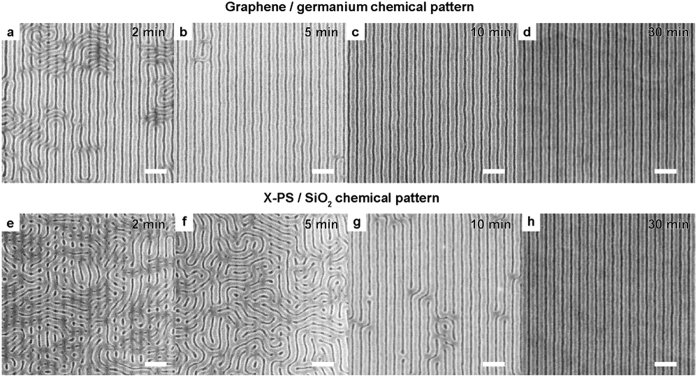
Assembly kinetics on graphene/germanium chemical patterns. (**a–h**) Assembled PS-b-PMMA after annealing at 250 °C on graphene/germanium chemical patterns (**a–d**) and X-PS/SiO_2_ chemical patterns (**e–h**) after 2 min (**a**,**e**), 5 min (**b**,**f**), 10 min (**c**,**g**), and 30 min (**d**,**h**). L_s_ is 78 nm. Scale bars are 200 nm.

Finally, PS-*b*-PMMA is directed to assemble on the graphene/germanium chemical patterns via thermal annealing (Fig. 1e). PS-*b*-PMMA is chosen for the directed assembly studies because it is among the most commonly studied and well understood systems used for block copolymer lithography^4^,^9^,^19^,^35^. The molecular weight of the PS-*b*-PMMA is 85k-*b*-91k g mol^−1^, corresponding to a bulk lamellar period (*L*_*0*_) of 78 nm. The graphene ribbon arrays are spin-coated with ~60 nm of PS-*b*-PMMA and the sample is annealed at 250 °C for 5–10 min in an inert N_2_ environment (O_2_ < 1 ppm and H_2_O < 1 ppm). Figure 2d shows that the PS-*b*-PMMA assembles into lamellae that closely follow the underlying graphene chemical pattern and are not disrupted by the germanium terracing. In Fig. 2, the period of the patterned graphene stripes, *L*_*s*_, is chosen to be equivalent to *L*_*0*_ = 78 nm to ensure that the PS-*b*-PMMA assembles under optimum conditions.

### Comparison of assembly kinetics

We compare the assembly kinetics of PS-*b*-PMMA on graphene/germanium templates and conventional chemical patterns based on X-PS (Fig. 2). The latter control samples consist of a patterned X-PS mat on a silicon oxide (SiO_2_) substrate. The graphene and X-PS films are patterned into similar line-space arrays with *L*_*s*_ = *L*_*0*_. The samples are spin-coated with PS-*b*-PMMA and annealed at 250 °C for 2–30 min to reach equilibrium. Directed assembly occurs more rapidly on the graphene-based chemical pattern than on the X-PS-based chemical pattern. Within 2 min, the block copolymer is largely ordered, following the underlying graphene template on >65% of the surface area (Fig. 2a); while the directed assembly remains local and is limited to short segments on the X-PS-based pattern (Fig. 2e). The block copolymer is well-assembled over >95% of the graphene/germanium chemical pattern after 5 min (Fig. 2b) and becomes completely ordered over the entire surface at 10 min (Fig. 2c). On the other hand, it takes 30 min to achieve the same degree of order on the X-PS-based chemical pattern (Fig. 2h). These results may indicate that the smooth, crystalline, rigid surfaces of graphene and germanium enable the polymers to diffuse more efficiently, resulting in faster assembly kinetics with fewer defects^22^.

### Directed assembly on chemical patterns with mismatched periodicity

**Figure 3:**
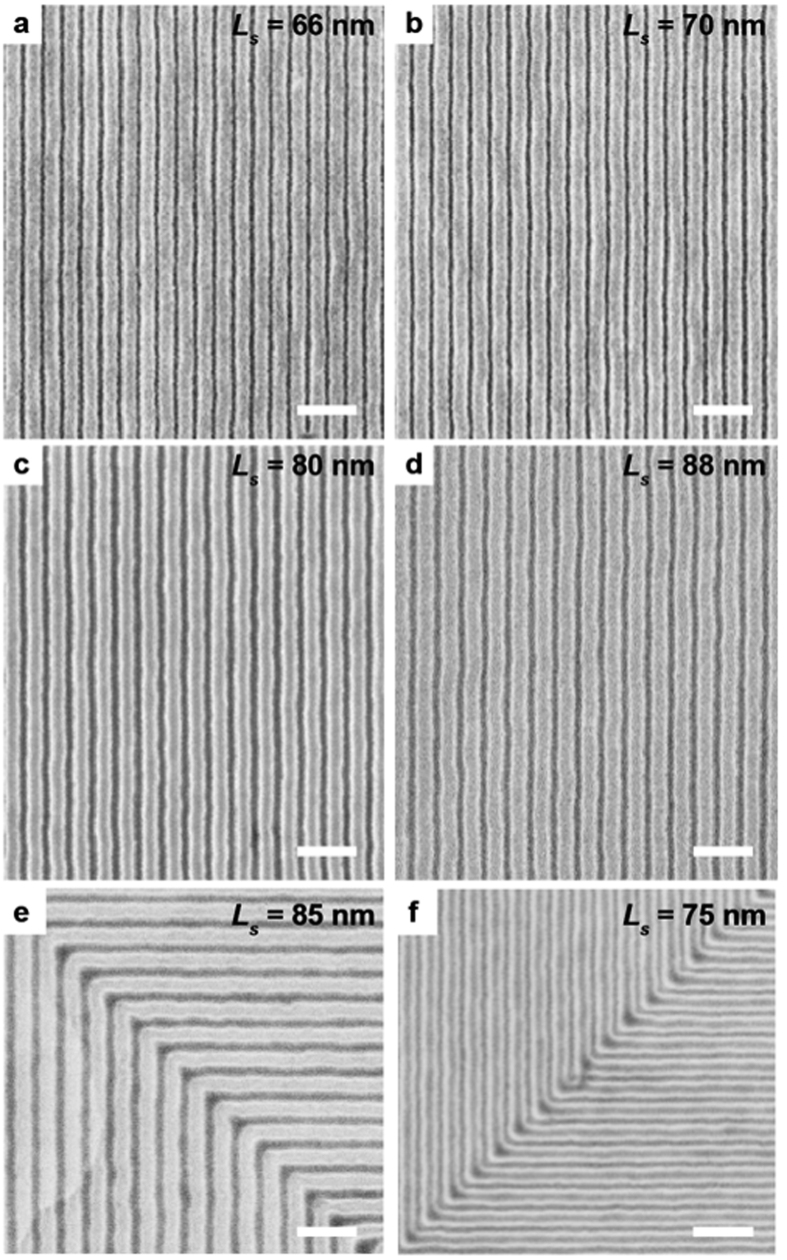
Directed assembly on incommensurate graphene/germanium chemical patterns. (**a–d**) Directed assembly of PS-*b*-PMMA on graphene/germanium chemical patterns with *L*_*s*_ of 66 nm (**a**), 70 nm (**b**), 80 nm (**c**), and 88 nm (**d**) corresponding to *L*_*s*_*/L*_*0*_ of 0.846, 0.897, 1.03, and 1.13, respectively. Scale bars are 200 nm. (**e**,**f**) Directed assembly of PS-*b*-PMMA on graphene/germanium chemical templates that are patterned into 90° bending structures with *L*_*s*_ of 85 nm (**e**) and 75 nm (**f**). Scale bars are 200 nm.

The ability of the block copolymer to conform, relative to *L*_*0*_, to the periodicity of the underlying chemical pattern during assembly is critical for applications such as bit patterned media^36^,^37^. PS-*b*-PMMA is assembled on graphene line arrays with *L*_*s*_ varying between 65 and 88 nm (Fig. 3a–d). The PS-*b*-PMMA is able to assemble on templates with 0.846 < *L*_*s*_*/L*_*0*_ < 1.13, allowing a broad commensurability window for designing the chemical pattern.

In addition to using an array of straight lines as the chemical pattern, directed assembly of PS-*b*-PMMA is also demonstrated on graphene/germanium chemical patterns with complex architectures (Fig. 3e,f). Bends with 90° angles, in which the line-to-line distance at the corner is 41% longer than *L*_*0*_, are patterned. Despite this large degree of incommensurability, the assembled PS-*b*-PMMA follows the graphene-based chemical pattern with 90° turns, as shown in Fig. 3e,f. In order to achieve such abrupt features using other types of chemical patterns, homopolymer/block copolymer blends are typically required to fill the gaps near the areas where the block copolymer domains bend^10^,^38^.

### Increased pattern resolution via density multiplication

**Figure 4:**
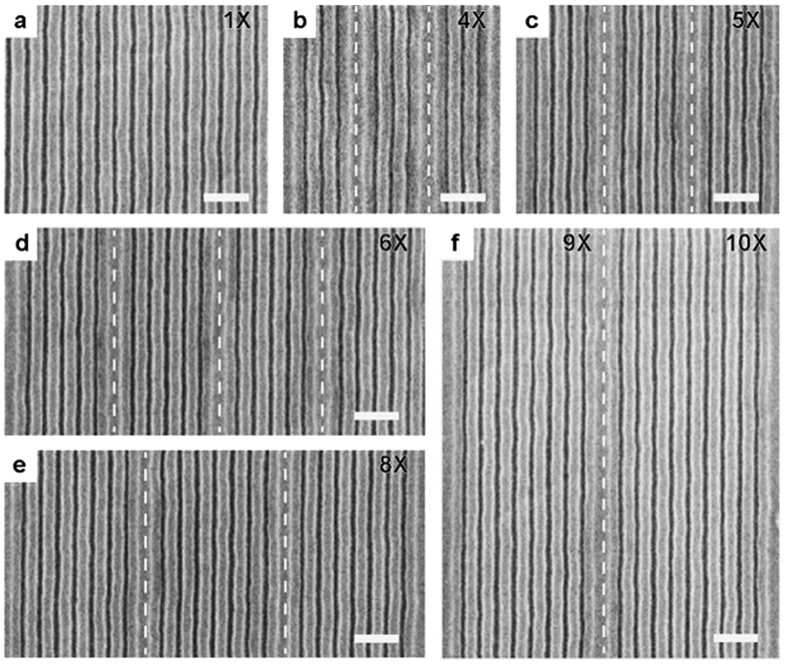
Density multiplication on graphene/germanium chemical patterns. (**a-f**) Directed assembly of PS-*b*-PMMA on graphene/germanium chemical patterns with density multiplication by a factor of 1 (**a**), 4 (**b**), 5 (**c**), 6 (**d**), 8 (**e**), and 9 and 10 (**f**). In **f**, the regions to the left and right of the dashed line are patterned with 9X and 10X density multiplication, respectively. Scale bars are 200 nm.

In order to enhance the resolution of the lithographically-defined template, assembly of block copolymers on graphene/germanium chemical patterns with density multiplication is explored (Fig. 4). The graphene is patterned into stripe arrays with *L*_*s*_ that are integer multiples, *n*, of *L*_*0*_ (where *n* = 1, 4, 5, 6, 8, 9, and 10). The line width of the graphene guiding stripes is chosen to be 1.5 *L*_*0*_ for *n* > 1 to clearly identify the underlying graphene stripes with SEM after directed assembly. The PS-*b*-PMMA is spin-coated onto the graphene ribbon array and the sample is annealed at 250 °C for 5–10 min to induce assembly. Despite the lack of guiding stripes for *n* periods of *L*_*0*_, the block copolymer segregates into regular domains between the graphene ribbons. On the wide graphene stripes, the PS and PMMA domains assemble into lamellae in which the bottom domains are nearly parallel to the surface, creating a U-shaped cross-section; this likely occurs because the PS preferentially wets the graphene surface when the width of a stripe is greater than *L*_*0*_, as previously reported on X-PS based chemical patterns^39^. The surface affinity of the graphene/germanium chemical pattern to PS-*b*-PMMA is further characterized, below. Density multiplication by a factor of 10 is achieved using the graphene chemical pattern (Fig. 4h), which is greater than the largest factor of 4 previously obtained using PS-*b*-PMMA with smaller molecular weight^18^,^39^. In addition to achieving density multiplication with 85k-*b*-91k PS-*b*-PMMA, the feature size can be further reduced to 12.5 nm by performing density multiplication using PS-*b*-PMMA with smaller molecular weight of 22k-*b*-22 k g mol^−1^, corresponding to *L*_*0*_ of 25 nm (Supplementary Fig. S2).

### Surface affinity measurements

Investigations of water contact angles and the wetting behavior of block copolymer films^40^ are conducted to gain insight into the mechanism governing the directed assembly (Supplementary Fig. S3). The water contact angle on graphene, germanium, PS, PMMA, and a random PS-PMMA (PS-*r*-PMMA) film is determined to provide an indirect measurement of their surface affinity towards each of the domains in PS-*b*-PMMA. The water contact angle on a continuous monolayer graphene film (81°) is similar to that of the PS film (91°). Furthermore, the contact angle on a bare germanium surface after plasma treatment (72°) is similar to that of the PMMA film (68°). These results suggest that PS and PMMA preferentially wet the graphene and germanium regions, respectively, during assembly.

The wetting behavior of the block copolymer domains on graphene and germanium is further verified with a hole/island experiment^40^. PS-*b*-PMMA (molecular weight of 22k-*b*-22 k g mol^−1^) thin films with thicknesses of 1.5 *L*_0_, 1.75 *L*_0_ and 2.0 *L*_0_ are spin-coated onto continuous graphene films after growth on germanium, and onto bare germanium surfaces after being treated with an oxygen plasma to etch the graphene. The samples are annealed at 190 °C for 1 h to induce self-assembly and AFM is used to resolve the equilibrium morphology of the block copolymer films. At 190 °C, PS has lower surface energy than PMMA, so it is energetically favored to wet the free surface. Thus, from the morphology of the self-assembled films, the surface affinity of graphene and germanium toward each block copolymer domain can be determined.

**Figure 5:**
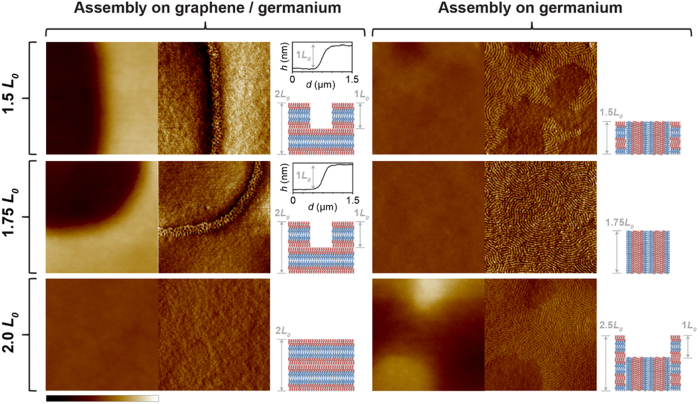
Determination of the surface affinity of graphene and germanium to the block copolymer domains with a hole/island experiment. Self-assembly behavior of 22k-*b*-22k PS-*b*-PMMA (*L*_0_ = 25 nm) on unpatterned graphene films on germanium (left half) and bare germanium (right half) surfaces. On each surface, an AFM height image, AFM phase image, and schematic of the assembled block copolymer domains are displayed side-by-side. These data are shown for PS-*b*-PMMA films with thickness of 1.5 *L*_0_ (top row), 1.75 *L*_0_ (middle row), and 2.0 *L*_0_ (bottom row). Line scans over a surface distance, *d*, for samples with PS-*b*-PMMA thickness of 1.5 *L*_0_ and 1.75 *L*_0_ on graphene show that the height, *h*, across the terrace boundary is 25 nm, ~1.0 *L*_0_. The corresponding schematics show the proposed arrangement of the block copolymer domains after assembly on graphene (preferential to PS) and germanium (slightly preferential to PMMA) surfaces. PS (PMMA) domains are red (blue). The height scale bars are 40 nm, the phase scale bars are 5°, and the size of each scan is 1.2 × 1.2 μm^2^.

The morphologies of the assembled PS-*b*-PMMA films are shown in Fig. 5. After assembly on the graphene surface (left column in Fig. 5), films with thickness of 1.5 *L*_0_ and 1.75 *L*_0_ have featureless terraces with step heights of 1.0 *L*_0_, whereas films with thickness of 2.0 *L*_0_ are flat and featureless. These results are consistent with symmetric wetting of the PS-*b*-PMMA, as shown in the assembly schematics in Fig. 5, and indicate that the graphene surface is preferential to PS domains. In contrast, after assembly on the germanium surface (right column in Fig. 5), fingerprint lamellae form at each thickness. Uniform fingerprint lamellae are obtained when the film thickness is 1.75 *L*_0_, which is consistent with assembly on a nearly neutral surface^40^. However, both featureless regions and fingerprint lamellae form when the film thickness is 1.5 *L*_0_ and 2.0 *L*_0_. The film is flat for the 1.5 *L*_0_ case, whereas the featureless regions are higher than the fingerprint regions for the 2.0 *L*_0_ case. As shown in the schematic of the assembled block copolymer domains in Fig. 5, this step height indicates that the germanium surface is slightly preferential to PMMA domains.

The difference in surface energy between the graphene and the germanium likely provides the chemical contrast needed for directed assembly; the PS and PMMA preferentially wet the graphene and germanium regions, respectively. This chemical contrast also provides a favorable template for directed self-assembly with density multiplication, as both the PS and PMMA domains can wet the nearly neutral germanium substrate to form well-ordered patterns, despite the lack of guiding stripes every L_0_^19^. Furthermore, the large degree of commensurability that is provided by the graphene guiding stripes may also be attributed to this chemical contrast, which can allow compensation of the energetic penalty when the block copolymers assemble on guiding stripes with *L*_S_ that deviates from *L*_0_^11^. Unlike the chemical affinity of X-PS, which can be altered by processing steps such as plasma trimming and brush backfilling^20^, the surface energy of graphene should be relatively unchanged during processing due to its high chemically inertness.

### Large-area directed assembly on graphene/SiO_2_ chemical patterns

The above results were obtained using graphene guiding stripes that are grown directly on germanium. The direct synthesis of the graphene chemical pattern on a target substrate is desirable for wafer-scale assembly of block copolymers because (1) CVD is an inherently scalable process, yielding uniform continuous graphene films over large areas that are only limited in extent by the size of the substrate or the size of the reaction chamber^41^, and (2) direct growth yields relatively pristine graphene films^42^,^43^, providing a clean, highly reproducible template on which to conduct directed assembly.

In order to demonstrate the generality of the directed assembly using graphene-based chemical patterns, we also transfer large-area monolayer graphene films grown via CVD onto alternative substrates using a sacrificial polymer support^28^, and subsequently conduct directed assembly experiments. SiO_2_ is chosen as the target substrate because it is preferential to the PMMA domains over the PS domains, and thus provides suitable chemical contrast and surface energetics with respect to graphene to direct the assembly of PS-*b*-PMMA. After transfer onto SiO_2_, the continuous graphene films are patterned into guiding stripes using electron-beam lithography and reactive ion etching, as described above. The 85k-*b*-91k PS-*b*-PMMA is directed to assemble into aligned lamellae on the graphene guiding stripes on SiO_2_ wafers with *L*_*s*_ of 78 nm (Supplementary Fig. S4). Notably, we also demonstrate directed assembly on these graphene/SiO_2_ chemical patterns over large areas (100 × 75 μm^2^) by patterning the guiding stripes using extreme ultraviolet (EUV) lithography. After directed assembly, the PS-*b*-PMMA is well-ordered over the entire patterned region, demonstrating that our approach is compatible with state-of-the-art planar lithographic techniques used for high-throughput patterning (Supplementary Fig. S4).

## Conclusion

In conclusion, we demonstrate that an atomically-thin monolayer graphene/germanium chemical pattern can be easily prepared and successfully used to direct the assembly of PS-*b*-PMMA. Graphene-based chemical patterns offer several unique advantages compared to conventional templates based on polymer mats and brushes. Specifically, graphene-based chemical patterns (1) provide faster assembly kinetics, (2) enable assembly on patterns with high degree of incommensurability, (3) allow assembly on irregular and complex templates, and (4) provide density multiplication by a factor of 10, even for relatively high molecular weight block copolymers. In future work, chemical contrast may be alternatively patterned by exposing the sample to chemical or plasma treatment, by forming a self-assembled monolayer, or by depositing a second two-dimensional atomic layer (e.g. by lateral growth, van der Waals epitaxy, or stacking). These graphene-based chemical patterns may enable the directed self-assembly of ultranarrow block copolymer domains for manufacturing of high resolution features, both in the semiconductor electronics industry and in high density magnetic media. This work also opens the door for directed assembly studies on chemical patterns based on the large library of two-dimensional materials.

## Methods

### Graphene synthesis

Ge(111) (Semiconductor Wafer, Inc) and Ge(110) (University Wafer) substrates are used to catalyze graphene growth, as described previously^42^,^43^. The germanium samples are sonicated in acetone and isopropyl alcohol for 15 min to clean their surfaces and then etched in deionized water at 90 °C for 15 min. The germanium substrates are placed into a horizontal tube furnace with a quartz tube inner diameter of 34 mm and the system is evacuated to ~10^−5^ torr. The chamber is filled to atmospheric pressure with a flow of 200 sccm of Ar (99.999%) and 100 sccm of H_2_ (99.999%). The samples are annealed for 30 min at 910 °C before introducing 4.6 sccm of CH_4_ (99.99%), which serves as the carbon precursor, to start the graphene synthesis. Growth occurs for 12 h to ensure complete graphene coverage on the germanium surface. The samples are rapidly cooled by sliding the furnace away from the growth region, which terminates growth.

### Characterization

AFM (Veeco MultiMode SPM) in tapping mode and SEM (Zeiss LEO 1530) are used to characterize the samples after graphene growth, after patterning of graphene into guiding stripes, and after directed assembly of PS-*b*-PMMA. The quality and thickness of graphene after growth and after patterning is assessed via Raman spectroscopy (Thermo Scientific DXRxi) using an excitation wavelength of 532 nm, power of 10 mW, and spot size of 0.6 μm. Contact angle measurements (Dataphysics OCA 15) are conducted using deionized water.

## Additional Information

**How to cite this article**: Chang, T.-H. *et al*. Directed self-assembly of block copolymer films on atomically-thin graphene chemical patterns. *Sci. Rep.* **6**, 31407; doi: 10.1038/srep31407 (2016).

## Supplementary Material

Supplementary Information
