# Supplementary material for: Directed self-assembly of block copolymer films on atomically-thin graphene chemical patterns
Source: Sci Rep. 2016 Aug 16;6:31407. doi: 10.1038/srep31407 (PMC4985650; doi:10.1038/srep31407)
Supplement: Supplementary Information [file srep31407-s1.pdf]

Directed self-assembly of block copolymer films on atomically-thin graphene chemical patterns

Tzu-Hsuan Chang[1†], Shisheng Xiong[2†], Robert M. Jacobberger[3†], Solomon Mikael[1], Hyo Seon Suh[2], Chi-Chun Liu[2,4], Dalong Geng[3], Xudong Wang[3], Michael S. Arnold[3], Zhenqiang Ma[1*], Paul F. Nealey[2*]

1. Department of Electrical and Computer Engineering, University of Wisconsin-Madison, Madison, Wisconsin 53706, United States
2. Institute for Molecular Engineering, University of Chicago, Illinois 60637, United States
3. Department of Materials Science and Engineering, University of Wisconsin-Madison, Madison, Wisconsin 53706, United States
4. IBM Albany NanoTech, Albany, New York 12203, United States

† These authors contributed equally.
* nealey@uchicago.edu, * mazq@engr.wisc.edu

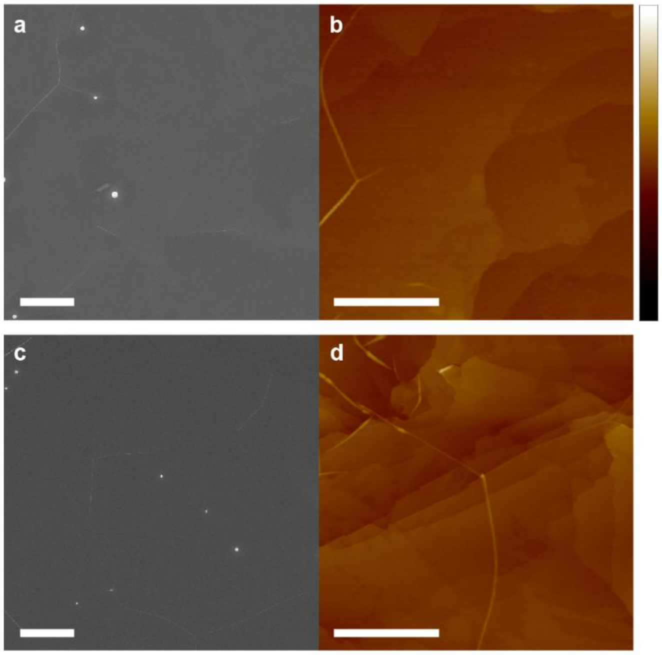

Supplementary Figure S1 Characterization of continuous monolayer graphene films on germanium. a-d, SEM (**a,c**) and AFM topographic (**b,d**) images of continuous monolayer graphene films after growth on Ge(111) (**a,b**) and Ge(110) (**c,d**). The scale bars in **a-d** are 1 μm and the height scale bars in **b,d,** are 20 nm. The faint lines running throughout the images are wrinkles in the graphene films that form while cooling the samples after growth.

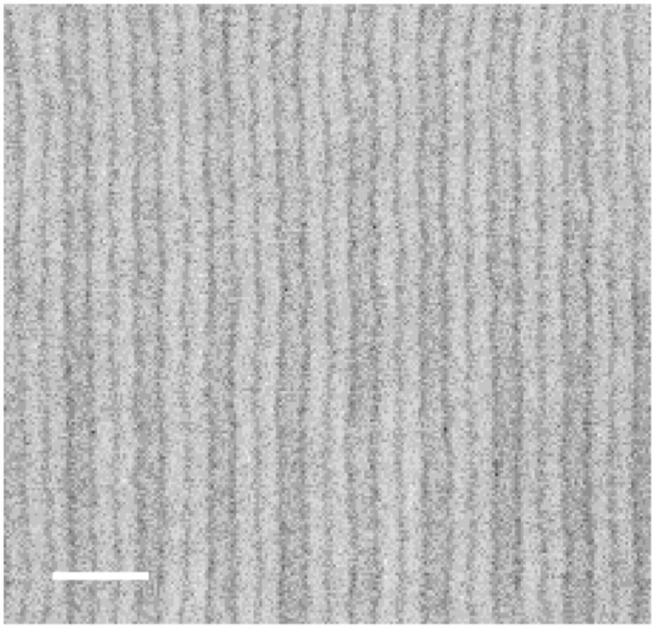

Supplementary Figure S2 Directed assembly of 22k-*b*-22k PS-*b*-PMMA with density multiplication. Density multiplication with 22k-*b*-22k PS-*b*-PMMA (L_0 = 25 nm) on a graphene/germanium chemical pattern. The graphene and germanium stripes are 75 and 25 nm wide, respectively. Density multiplication by a factor of 3 is achieved to obtain a feature size of 12.5 nm. Scale bar is 100 nm.

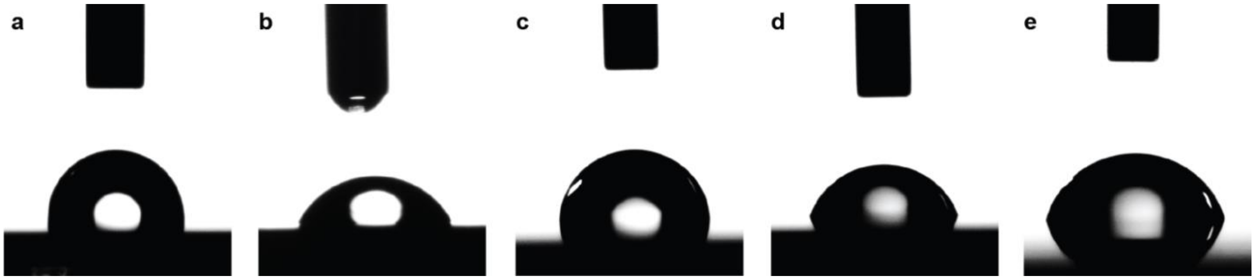

Supplementary Figure S3 Water contact angle measurements. a-e, Water contact angles measurements on a graphene surface after solvent rinsing in chlorobenzene and toluene and annealing at 350 °C at ~10^{-5} torr for 2 h to remove residue and contamination introduced during processing (**a**), on a bare germanium surface after oxygen (10 sccm) plasma etching at 50 W and 10 mtorr for 1-2 s to remove graphene (**b**), on a PS film (**c**), on a PMMA film (**d**), and on a PS-*r*-PMMA random brush neutral film consisting of 57% PS by weight composition (**e**). The results are summarized in Supplementary Table 1, below.

Supplementary Table S1

	**Graphene**	**Germanium**	**PS**	**PMMA**	**PS-*r*-PMMA**
**Contact angle (degrees)**	81	72	91	68	72

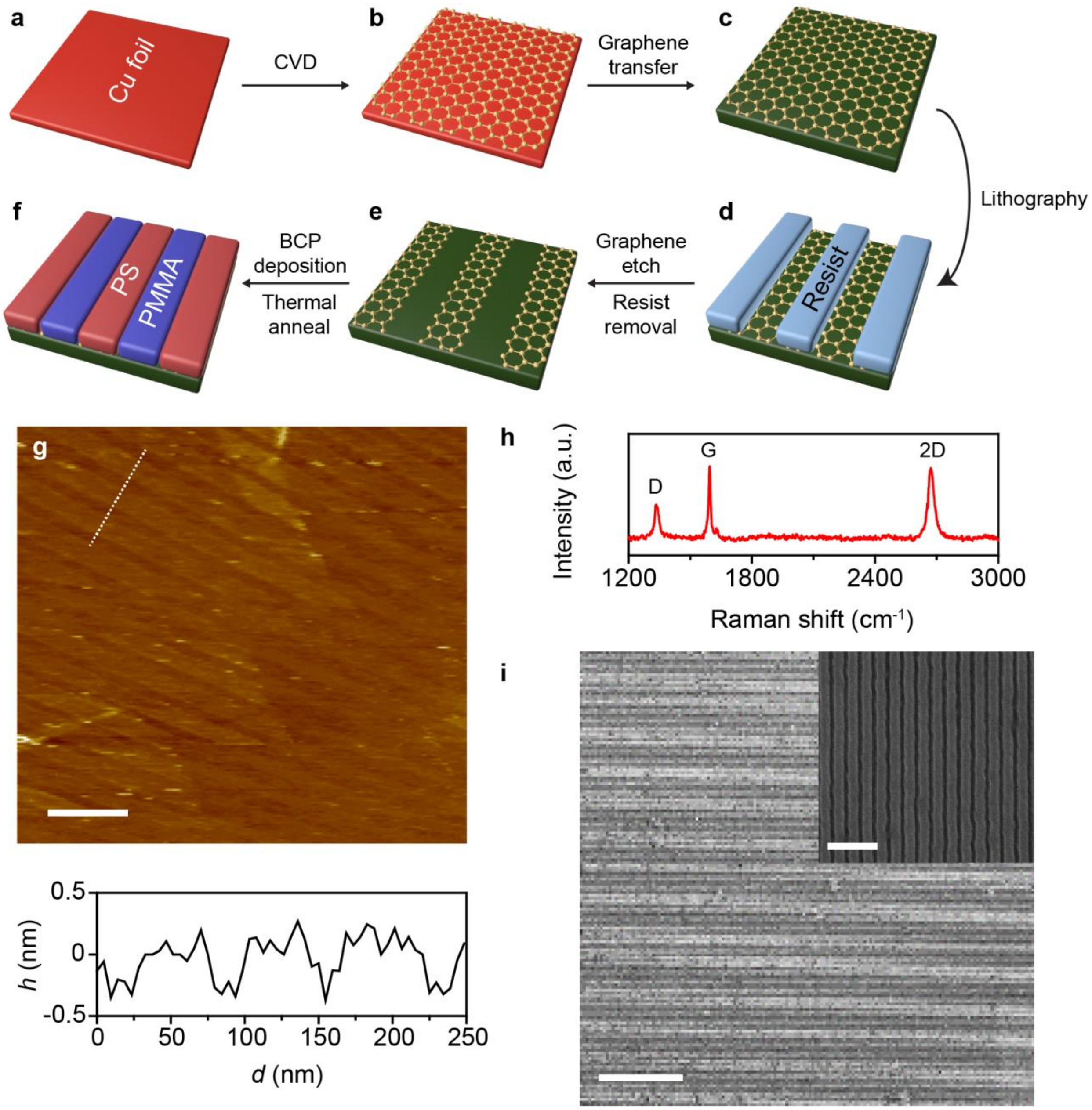

Supplementary Figure S4 Directed assembly of PS-*b*-PMMA on graphene/SiO_2 chemical patterns. a-f, Process flow of the directed assembly of block copolymers on graphene/SiO_2 chemical patterns. Graphene is grown on copper foil via CVD (**a,b**). The graphene is transferred onto SiO_2. PMMA is used to provide mechanical support and the copper is etched with $FeCl_3$. The graphene membrane is subsequently transferred onto SiO_2 and the PMMA support is removed in acetone (**c**). Resist is spin-coated onto the graphene surface and patterned into stripe arrays with electron-beam lithography or extreme ultraviolet lithography (**d**). The exposed graphene is etched using a reactive oxygen ion plasma and the resist is removed via solvent rinsing and thermal annealing, resulting in a graphene/SiO_2 stripe array (**e**). The block copolymer is spin-coated on the chemical pattern and thermally annealed to direct assembly (**f**). **g,** AFM topographic image of patterned graphene stripes on SiO_2 with L_s of 78 nm. Scale bar is 200 nm. Height (h) profile along the white line shows that the graphene step height is ~0.5 nm. **h,** Raman spectrum from a graphene stripe array on SiO_2. **i,** Low-magnification SEM image of 85k-*b*-91k PS-*b*-PMMA after directed

assembly on a graphene/SiO_2 chemical pattern with L_s of 78 nm that was patterned over 100 x 75 μm^2 using extreme ultraviolet lithography. Scale bar is 5 μm. An interference pattern forms due to the well-ordered block copolymer domains. The inset shows a high-magnification SEM image of the assembled PS-*b*-PMMA from a representative region within the 100 x 75 μm^2 area. Inset scale bar is 250 nm.
